# Long-Term Outcome of Patients Followed by Nephrologists after an Acute Tubular Necrosis Episode

**DOI:** 10.1155/2012/361528

**Published:** 2012-11-27

**Authors:** G. A. Brito, A. L. Balbi, J. M. G. Abrão, D. Ponce

**Affiliations:** Botucatu School of Medicine, University São Paulo State (UNESP), Distrito de Rubiao Junior, 18608000 Botucatu, SS, Brazil

## Abstract

Aims of our study were to describe the long-term survival in patients surviving an acute tubular necrosis (ATN) episode and determine factors associated with late mortality. We performed a prospective cohort study that evaluated the long-term outcome of 212 patients surviving an ATN episode. Mortality at the end of followup was 24.5%, and the probability of these patients being alive 5 years after discharge was 55%. During the followup, 4.7% of patients needed chronic dialysis. Univariate analysis showed that previous CKD (*P* = 0.0079), cardiovascular disease (*P* = 0.019), age greater than 60 years (*P* < 0.0001), and higher SCr baseline (*P* = 0.001), after 12 months (*P* = 0.0015) and 36 months (*P* = 0.004), were predictors of long-term mortality. In multivariate analysis, older age (HR = 6.4, CI 95% = 1.2–34.5, *P* = 0.02) and higher SCr after 12 months (HR = 2.1, 95% CI 95% = 1.14–4.1, *P* = 0.017) were identified as risk factors associated with late mortality. In conclusion, 55% of patients surviving an ATN episode were still alive, and less than 5% required chronic dialysis 60 months later; older age and increased Scr after 12 months were identified as risk factors associated with late death.

## 1. Introduction

Acute tubular necrosis(ATN)is themost frequent cause ofacute kidney injury(AKI)in hospitalized patients [1–4]. Despite recent progress in the management of AKI patients, mortality remains high,around 50%, and reaches 80% in septic and dialysis patients [4–9].

Recent studies have claimed thatlong-term mortality in patients afterATN varied from 15% to 72% depending on the setting and time period studied [10–14].When evaluating the long-term outcome of ATN, not only survival but recovery of renal function (RF) is a central issue. Unfortunately, data on long-term functional outcomes are still controversial [12, 13]. Previous studies reported good recovery of RF after ATN [12, 13], while other recent studies described a high rate of dialysis-dependent patients [10, 15–17]. Actually, the long-term effects of ATN are not well understood and the factors associatedwith survival andrecovery of RF are not yetcompletely defined.

The aims of ourstudy were to describe the long-termsurvival andrecovery of RF in patients surviving an ATN episode and being followed by the same nephrologist and determine factors associated withlong-term mortality.

## 2. Patients and Methods

### 2.1. Population, Study Periods, and Definitions

This was a prospective cohort study performed at a single centre with ATN patients treated and followed up by the AKI Group of Botucatu School of Medicine, University of Sao Paulo State, Brazil, from October 2004 to May 2011. ATN patients discharged alive and followed by nephrologists for at least one year comprised the population analysed. 

Followup was every three months during the first year of followup, every six months in the second year and then annually thereafter.Patients were evaluated after 12, 36, and 60 months for survival and recovery of RF.

We excluded patients younger than 18 years, with other etiologies of AKI, with advanced chronic kidney disease (CKD) (glomerular filtration rate <30 mL/min as estimated by MDRD), those with renal transplantation and pregnant women.

AKI was defined as elevation of serum creatinine (SCr) based on the criteria of AKIN [18]. ATN was diagnosed as a history of prolonged and profound hypotension, severe nephrotoxic drug overdose, or excess endogenous nephrotoxic pigments (hemoglobinuria, myoglobinuria). Diagnosis was based on clinical history, results of physical examination, relevant blood tests, urinalysis (microscopic examination of urinary sediment), a fractional excretion of sodium that exceeded 1%, and the findings on renal ultrasonography.

Severity of ATN was classified as stage I, II, or III according to the ratio between the highest SCr found during the AKI episode and the baseline SCr: Stage I, SCr ≤ 2, Stage II, SCr 2 to 3, and Stage III, SCr > 3. All patients who needed dialysis were classified as stage III [18]. 

Variables recorded during ATN admission were sex and age, comorbid conditions (cardiac, hepatic and tumours, diabetes mellitus, hypertension and CKD), intensive care unit (ICU) admission, ATN etiology (septic, ischemic, nephrotoxic or mixed), SCr levels (baseline, at the moment of AKI diagnosis, the highest value during admission and at discharge), and the need for and type of dialysis (hemodialysis or peritoneal). 

Acute Tubular Necrosis-Index Severity Score (ATN-ISS) was calculated at the moment of AKI diagnosis [7]. 

Baseline SCr was defined as the lowest SCr value in the last 6 months before AKI or, for those without this measurement, the lowest value achieved during hospitalization in the absence of dialysis [15]. 

Recovery of RF at hospital discharge was classified as total (SCr returned to baseline levels), partial (SCr remained higher than baseline, but not requiring dialysis), or absent (patient still needed dialysis). 

RF during the followup was classified as recovery of RF or progression to CKD, defined as a 10% reduction in GFR calculated according to the MDRD [19]. Previous CKD was defined as GFR lower than 60 mL/min.

ATN patients were divided into two groups, survivors (SG) and nonsurvivors (GNS), and compared for clinical characteristics and recovery of RF. Patients missing followup were excluded from this analysis. 

Informed consent was obtained from study participants or their legal counsel. The study was approved by the local ethics committee on 2 August 2010.

### 2.2. Statistical Analysis

Continuous variables were expressed as mean ± SD or medians and compared using the Student's *t*-test or Mann-Whitney test, as appropriate. Categorical variables were expressed as proportions and compared with the chi-squared test.

To identify risk factors associated with death, we performed univariate analysis using a log rank test, based on clinical and laboratory variables that could have an effect on mortality, according to data in the literature.

To analyse the evolution of RF, we used logistic regression models for repeated measures at time zero (discharge) and a proportional odds model for repeated measurements during followup (12, 36, and 60 months).

Survival curves were estimated by the Kaplan-Meier method, and differences between curves assessed with the log-rank test. Multivariate survival analysis was performed via the Cox model using backward variable selection, with the exit criteria set at *P* < 0.25. Statistical analyses were conducted using SPSS 13.0.

## 3. Results

**Figure 1:**
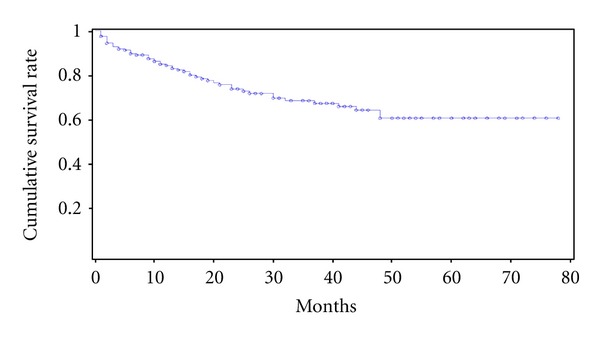
Patients survival curves after acute tubular necrosis episode.

From an initial population of 950 ATN cases, 686 (72.2%) were in the ICU, average ATN-ISS was 0.61 (0.29–0.78), 590 (62.1%) died during hospitalization, and 148 (15.5%) were excluded because of unknown outcomes. Two hundred and twelve surviving patients (22.3%) constituted the population analysed. The patients' characteristics are shown in Table 1. Average age of the population studied was 59.2 years (48–71), 62.7% were male, 64 (30.2%) were in the ICU, CKD was present in 39.1% of the patients, and 38.6% had cardiovascular diseases.Of all the patients followed, 23.5% required dialysis during hospitalization. Forty-one patients (19.3%) were lost during followup so the follow-up rate was 81.7%. Mortality at the end of followup was 24.5%. Patients were followed between 2 and 64 months. Fifty percent of the patients had a followup averaging 24.4 months (9–39). The probability of these patients being alive 5 years after discharge was 55% (Figure 1). Causes of death were available for 41 nonsurvivors (78.9%). The main causes of death were diseases of the circulatory system (36.5%), neoplasms (26.8%), and diseases of the respiratory system (17%).

At hospital discharge, total recovery of RF had occurred in 36.7% of patients, while 0.9% needed dialysis. During the follow-up period, 4.7% of patients needed chronic dialysis. At the first assessment, the GFR according to MDRD was 52 ± 11 mL/min, and after 12, 36, and 60 months of followup, it was 58.1 ± 17.2 mL/min (*P* = 0.04 compared tothe first assessment), 57 ± 15.2 mL/min (*P* = 0.07) and 57.2 ± 14.2 mL/min (*P* = 0.09), respectively. 

Patients were evaluated for progression to CKD after 12 months of followup.Among patients with GFR > 60 mL/min, 8.7% progressed to CKD stages 4 or 5; the same occurred in 22.8% of patients with GFR < 60 mL/min (*P* < 0.01).Only patients with GFR < 60 mL/min at the first assessment progressed to end-stage renal disease (ESRD) after 12 months of followup.

During their hospital stay, 50 patients (23.5%) required dialysis; long-termsurvival andrecovery of RF in these patients were similar to the general pool of patients surviving an ATN episode. Mortality at the end of followup was 19.1%, and these patients were followed between 2 and 61 months. Fifty percent of the patients had a followup of 28.4 months (8–37). The probability of these patients being alive 5 years after discharge was 51%. At hospital discharge, 2.4% needed dialysis, and after 12 months of followup 12% of patients progressed to CKD stages 4 or 5 and none of the patients needed chronic dialysis.

After we excluded the patients who were lost to followup (19.3%), the others were divided into two groups: survivors (GS) and nonsurvivors (GNS).Table 2 compares them with regard to clinical aspects, laboratory values, and outcome. There was a predominance of males in both groups (GS = 61.3% versus GNS = 61.5%, *P* = 0.97).There was a difference between the two groups in age (GS = 56.4 ± 16.1 versus GNS = 69.3 ± 12.2 years, *P* < 0.001), presence of CKD before the ATN episode (GS = 37% versus GNS = 55.7%, *P* = 0.008),cardiovascular disease (GS = 37% versus GNS = 57.7%, *P* = 0.02), baseline SCr (GS = 1.3 versus GNS = 1 mg/dL, *P* = 0.0003), sepsis as main diagnosis (GS = 25.6% versus GNS = 44.2%, *P* = 0.001), and follow-up time (GS = 18 versus GNS = 10.5 months, *P* = 0.04). The GS and GNS groups were similar in type of admission, ATN etiology, ATN severity, and creatinine peak.

Univariate analysis showed that previous CKD (log rank = 9.14, *P* = 0.0079), cardiovascular disease (log rank = 8.18, *P* = 0.019), age greater than 60 years (log rank = 1.05, *P* < 0.0001), and higher SCr baseline (log rank = 2.18, *P* = 0.001) after 12 (log rank = 1.43, *P* = 0.0015) and 36 (log rank = 1.58, *P* = 0.004) months of followup were predictors of long-term mortality.Table 3 shows these results.

**Figure 2:**
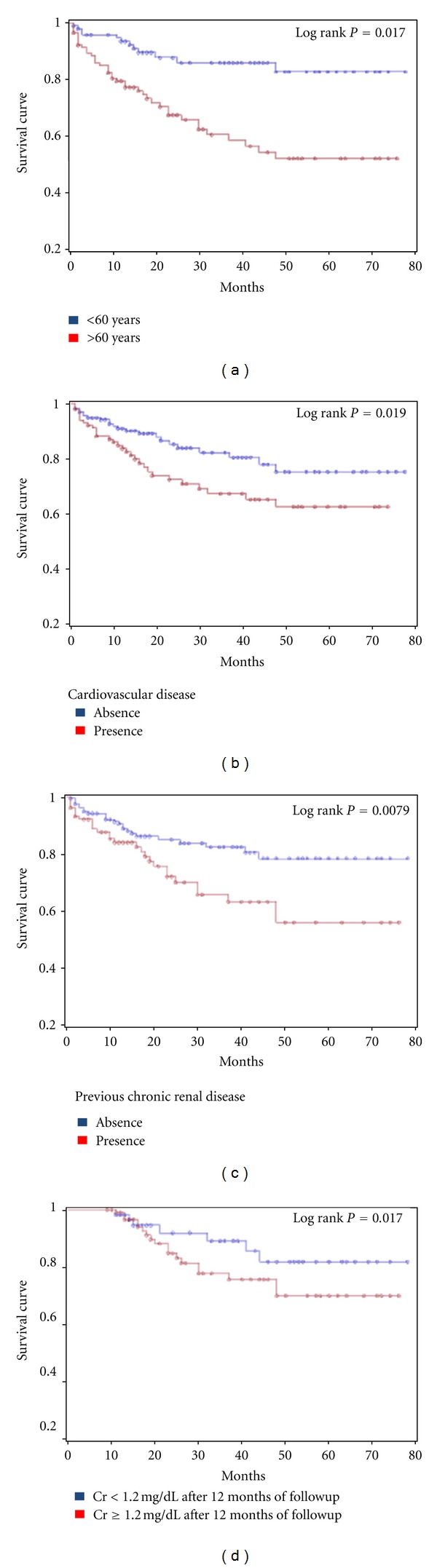
Kaplan-Meier survival curves according to (a) age; (b) the presence or absence of cardio-vascular disease; (c) the presence or absence of previous chronic renal disease; (d) serum creatinine after 12 months ≥ or <1.2 msg/dL.

Figure 2 shows the survival curves for categorical variables, including previous CKD and cardiovascular disease, age greater than 60 years, and SCr after 12 months of followup (higher or lower than 1.2 mg/dL).Patients without previous CKD, younger than 60 years, without cardiovascular disease and SCr < 1.2 mg/dL after 12 months of followup showed better survival at the end of followup.

Upon multivariate analysis, risk factors associated with long-term mortality included old age (HR = 6.4, CI 95% = 1.2–34.5, *P* = 0.02) and higher SCr after 12 months of follow-up (HR = 2.1, 95% CI 95% = 1.14–4.1, *P* = 0.017), as shown in Table 4. There were no differences in survival in relation to sex, ATN etiology, ATN-ISS, need for dialysis, presence or absence of oliguria, or the degree of renal function at discharge.

## 4. Discussion

This study aimed to describe the long-term patients' outcome on survival and progression to CKD after an episode of ATN and identify risk factors associated with death.

In this prospective cohort study, 950 ATN patients were evaluated in-hospital, 590 (62.1%) died during hospitalization, and 212 (22.3%) had hospital discharge and were followed by the same nephrologist. Mortality rate during followup was 24.5%. Of patients surviving an ATN episode, 55% were still alive and 4.7% had progressed to ESRD requiring dialysis after 60 months. 

These results are similar to those described in the literature previously. Liãno et al. cols [12] evaluated 177 patients surviving an ATN episode and 50% were alive 10 years later. No patient required dialysis at hospital discharge and only two patients progressed to chronic dialysis during the follow-up period. Schiffl and Fischer [20] followed up 226 patients with severe AKI, (defined as need for dialysis) and absence of previous CKD and the observed mortality rate was 75% after 5 years and recovery of RF was 86% (in survivors). No patient was dependent on dialysis at hospital discharge, and 5% required dialysis after 5 years of followup. Uchino et al. [21], in BEST study, showed that 13.8% of patients were dialysis dependent at discharge hospital, which can be considered higher than our results. However, Uchino et al. [21] followed only AKI patients that required dialysis during hospitalization, while we followed up patients who were treated and nontreated with dialysis.

Data related to the progression to CKD are conflicting. Some studies have shown that patients after an ATN episode had favourable outcomes with low progression to CKD and need for chronic dialysis. However, these and other similar studies [12, 13, 18] had several difficulties, including several different definitions of AKI, great variability in the long-term follow-up period of the patients studied (ranging from 6 to 372 months), small numbers of patients, and different definitions of progression to CKD and recovery of RF. Recently new studies have shown that patients surviving an ATN episode are at increased risk of progression to advanced CKD (Stages 4 and 5) [10, 15–17].

The US Renal Data System 2010 Annual Data Report indicates that survivors of AKI are at risk of developing ESRD within the following year. This risk increases from less than 1% for those without previous CKD to 5% for those with previous CKD [22].

Our data indicated that, after an ATN episode, patients showed favourable renal function outcomes in the long-term and the most important changes (recovery or progression) occurred within the following year. 

GFR remained stable in 41.3% of patients. The risk of progression to CKD was higher in patients with previous CKD (GFR < 60 mL/min) than those without it (GFR > 60 mL/min). Less than 9% of patients without previous CKD progressed to CKD Stages 4 or 5, while 23% of patients with previous CKD progressed to CKD Stages 4 or 5. These results are similar to that found by Amdur et al. [15], where 20% of those with ATN progressed to CKD stage 4 or 5. Need for chronic dialysis was present in 4.7% after 60 months of followup, and all patients who progressed toward ESRD had previous CKD.

Similar results were recently observed by Macedo et al. [23] showing that 4 patients (4.7%) progressed to ESRD; however, only one (1.1%) did so within the first year after AKI.

Our data agree with Wu et al. [24] that evaluated 9425 patients after severe AKI (with need for dialysis). Patients with previous CKD presented higher risk of progression to ESRD when compared with patients without previous CKD (17.8 × 0.15 patients/year). The risk of progression to ESRD was also higher when patients with previous CKD did not have total recovery of RF at hospital discharge (RR = 212.7, 95% CI 105.5–428.8, *P* < 0.001).

Thakar and colleagues [25] studied patients with diabetes and risk of progression to CKD after ATN episodes. It was observed that an episode of AKI in diabetic patients compared with diabetic patients without ATN was associated with progression to CKD Stage 4 (RR = 3.56, 95% CI 2.76–4.61), and that each new episode of AKI led to increased risk (RR = 2.02, CI = 1.78–2.3).

Recent studies have shown that ATN episodes may be independently associated with risk of death in the long term [10, 26]. Hsu et al. [27] evaluated 2155 patients after an ATN episode concerning late mortality. Patients were followed up for 10 years, and AKI was identified as an independent risk factor for death (OR = 1.24, 95% CI = 1.07 to 1.44, *P* < 0.01) compared with patients without ATN. Lo et al. [10] evaluated patients without CKD after a severe ATN episode and also observed that ATN was associated with increased risk of death after 8 years of followup (OR = 2.3, 95% CI = 1.8 to 3.0).

A recent study evaluated patients with previous CKD and risk of death after AKI episodes. It was observed that an episode of ATN in patients with previous CKD compared with patients without ATN was associated with increased risk of death (OR = 1.3, 95% CI = 1.04–1.64) [27]. Lafrance and Miller [28] suggest that even after adjusting for CKD, an ATN episode is associated with increased risk of death in the long term.

In the present study, the nonsurvivors compared to long-term survivors after an ATN episode had more ATN associated with sepsis, age, and prevalence of previous CKD and cardiovascular disease. Ponte et al. [13], analysing 413 patients after ATN found conflicting results, suggesting the presence of sepsis or systemic inflammatory response syndrome was associated with high initial mortality, but with better long-term survival.

In this study, multivariate analysis identified age and increased SCr after 12 months of followup as risk factors associated with late mortality. These results agree with data reported in the literature. Survival curves were better in patients without previous CKD, nonelderly patients (<60 years), without cardiovascular disease and with SCr less than 1.2 mg/dL after 12 months.

Similar results were shown by Schiffl and Fischer [20], who identified age, surgical admissions, comorbidities, and partial recovery of RF at hospital discharge as predictors of long-term mortality after severe AKI. Liãno et al. [12] evaluated the long-term survival of patients without previous CKD after one ATN episode and identified age, comorbidity, and male sex as predictors of late mortality.

Our study has several limitations and cannot be generalized to ATN patients as a whole. It is a one-centre prospective study, there is no control group similar to the studied population, and the follow-up time was short and most of the patients were followed up for less than 60 months. It is the first study in which followup was made by the same nephrologist during hospitalization and long-term outpatient followup, who was well aware of the possibility of ATN progression to CKD and tried to preserve renal function. Thus, our results may indicate a better than usual long-term outcome after ATN: only 4.7% progressed to ESRD after 5 years. 

In conclusion, this study showed 55% of patients surviving an ATN episode were still alive 60 months later and less than 5% required chronic dialysis; age and increased Scr after 12 months of follow-up were identified as risk factors associated with late death.

Finally, it is increasingly recognized that ATN should not only be considered an acute syndrome, but it can be a risk condition for progression of CKD and late mortality. Because of this the survivors of ATN deserve a careful and long-term medical followup.

## Figures and Tables

**Table 1:** Characteristics of the survivor patients after acute tubular necrosis episode.

Patients	*n* = 212
Male gender (%)	133 (62.7)
Age (years)	59.2 (48–71)
ATN-ISS	0.33 (0.18–0.43)
Previous chronic renal disease (%)	83 (39,1)
Hypertension (%)	73 (34.4)
Cardio-vascular disease (%)	142 (66.9)
Diabetes (%)	73 (34.4)
ATN etiology (%)	
(i) Ischaemic	57 (26.9)
(ii) Nephrotoxic	46 (21.7)
(iii) Mix	50 (23.6)
(iv) Septic	59 (27.8)
In-hospital	
Type of admission (%)	
(i) Surgical	54 (25.5)
(ii) No surgical	158 (74.5)
ICU admission (%)	64 (30.2)
ATN severity (%)	
(i) stage I	55 (25.9)
(ii) stage II	48 (22.6)
(iii) stage III	109 (51.5)
SCr peak (mg/dL)	5.1 (2.6–6.7)
Dialysis (%)	50 (23.5)
Recovery of renal function (%)	
(i) Total	78 (36.7)
(ii) Partial	132 (62.4)
(iii) Absence	2 (0.9)
Followup	
Time of followup (months)	24.4 (9–39)
MDRD (mL/min)	
First evaluation	52 ± 11
After 12 months	58.1 ± 17.2
After 36 months	57 ± 15.2
After 60 months	57.2 ± 14.2
Need for late dialysis (%)	10 (4.7)
Mortality (%)	52 (24.5)
Lost of follow-up	41 (19.3)

ATN-ISS: acute tubular necrosis-individual severity score; data showed in %, means ± sd or median (q1–q3).

**Table 2:** Comparative analysis of survivor (SG) and non-survivor (NSG) patients after an episode of ATN according to clinical and laboratory characteristics and outcome.

	SG (*n* = 119)	NSG (*n* = 52)	*P *
Male gender (%)	61.3	61.5	0.97
Age (years)	56.3 ± 16.1	69.3 ± 12.2	<0.0001
ATN-ISS	0.26 (0.15–0.45)	0.28 (0.19–0.43)	
Cardio-vascular disease (%)	37	57.7	0.02
Previous chronic renal disease (%)	37	55.7	0.008
Diabetes (%)	33.6	42.3	0.34
Hypertension (%)	70	69.2	
Sepsis (%)	25.6	44.2	0.001
Type of admission (%)			
(i) Surgical	32.7	28.8	
(ii) No surgical	67.3	71.2	
ICU admission (%)	31.9	28.8	
SCr baseline (mg/dL)	1 (0.8–1.3)	1.3 (1–1.5)	0.0003
SCr after 12 months	1.3 (1–1.7)	1.6 (1.1–1.9)	0.08
SCr peak (mg/dL)	4.3 (2.6–6.9)	3.7 (2.4–6.4)	
ATN etiology (%)			
(i) Ischaemic	26.9	39.6	
(ii) Nephrotoxic	22.7	18.9	
(iii) Mix	23.5	18.9	
(iv) Septic	26.9	22.6	
ATN severity (%)			
(i) Stage I	23.6	25	
(ii) Stage II	24.5	28.8	
(iii) Stage III	50.9	40.2	
Followup (months)	18 (11–44.5)	10.5 (3–20.5)	0.04
Dialysis in-hospital (%)	26.0	17.3	0.20
Recovery of renal function (%)	35.3	36.5	0.83

SCr: serum creatinine.

Data showed in %, means ± sd or median (q1–q3).

**Table 3:** Univariate analysis of risk factors associated with late mortality of patients after an episode of acute tubular necrosis.

Variable	log rank	*P *
Previous chronic renal disease	9.14	0.0079
Cardiovascular disease	8.18	0.019
Diabetes	3.19	0.34
Hypertension	1.40	0.67
Tumours	2.25	0.22
Dialysis in-hospital	3.91	0.2
Total renal recovery	0.70	0.83
Partial renal recovery	0.51	0.88
Age (per 1 year)	1.05	<0.0001
SCr after 12 months (per 0.1 mg/dL)	1.43	0.0015
SCr after 36 months (per 0.1 mg/dL)	1.58	0.004
SCr baseline (per 0.1 mg/dL)	2.18	0.001

SCr: serum creatinine.

**Table 4:** Multivariate analysis of risk factors associated with late mortality of patients after an episode of acute tubular necrosis.

Variable	HR	IC 95%	*P *
Cardiovascular disease	1.77	0.94–2.1	0.19
Previous chronic renal disease	1.98	0.91–2.6	0.23
Tumours	1.64	0.88–1.8	0.31
Age (per 1 year)	6.4	1.2–34.5	0.02
SCr after 12 months (per 0.1 mg/dL)	2.1	1.14–4.1	0.017
SCr after 36 months (per 0.1 mg/dL)	1.87	0.83–2.12	0.36

SCr: serum creatinine.
